# Review of Literatures: Physiology of Orofacial Pain in Dentistry

**DOI:** 10.1523/ENEURO.0535-20.2021

**Published:** 2021-04-26

**Authors:** Nattapon Rotpenpian, Pankeaw Yakkaphan

**Affiliations:** 1 Department of Oral Biology and Occlusion, Faculty of Dentistry, Prince of Songkla University, Songkhla, Thailand 90110; 2 Department of Oral Diagnostic Science, Faculty of Dentistry, Prince of Songkla University, Songkhla, Thailand 90110

**Keywords:** dentistry, neurophysiology, orofacial pain, pain, sensation, sensory function

## Abstract

The objective of this review of the literature is to summarize the physiology of orofacial pain in dentistry, particularly physiology of the pain pathway and molecular mechanisms on pathophysiology of pain, on account of new insights into classification of orofacial pain related diseases. This article will also focus on possible mechanisms of neuropathic orofacial pain which is distinguished from other types of pain.

## Introduction

Pain is a sensory modality that is an unpleasant and emotional experience (Pak et al., 2018). Painful sensation is subjective and biologically useful, necessary for survival, being a warning sign and response of damaged tissue in the body (John, 1990; Rivera-Morales, 1986). Pain in the facial area is the most common reason which brings patients to see a dentist (Piovesan et al., 2003). Orofacial pain is pain associated with the hard and soft tissues of the head, face, and oral cavity (Sessle, 1987). There are diverse and several mechanisms related to this pathology (Sessle, 1987). Therefore, orofacial pain physiology should be elucidated and applied to clinical practice in the future. This review will define the physiology of orofacial pain and classification of orofacial pain in dentistry. In this review, the strategy for the search in terms of pain, pathophysiology of pain, orofacial pain, dental pain, nociceptive pain, neuropathic pain, pain pathway, and the publication year is 1980–2021.

## Physiology of the Orofacial Pain Pathway

The orofacial region is composed of the oral cavity (teeth, gingiva, and oral mucosa), face, jaw bone, and temporomandibular joint (Messlinger and Handwerker, 2015). Physiology of orofacial pain pathways includes primary afferent neurons, pathologic changes in trigeminal ganglion, brainstem nociceptive neurons, and higher brain function regulating orofacial nociception.

## Primary Afferent Neurons and Pathologic Changes in Trigeminal Ganglion

**Figure 1.**
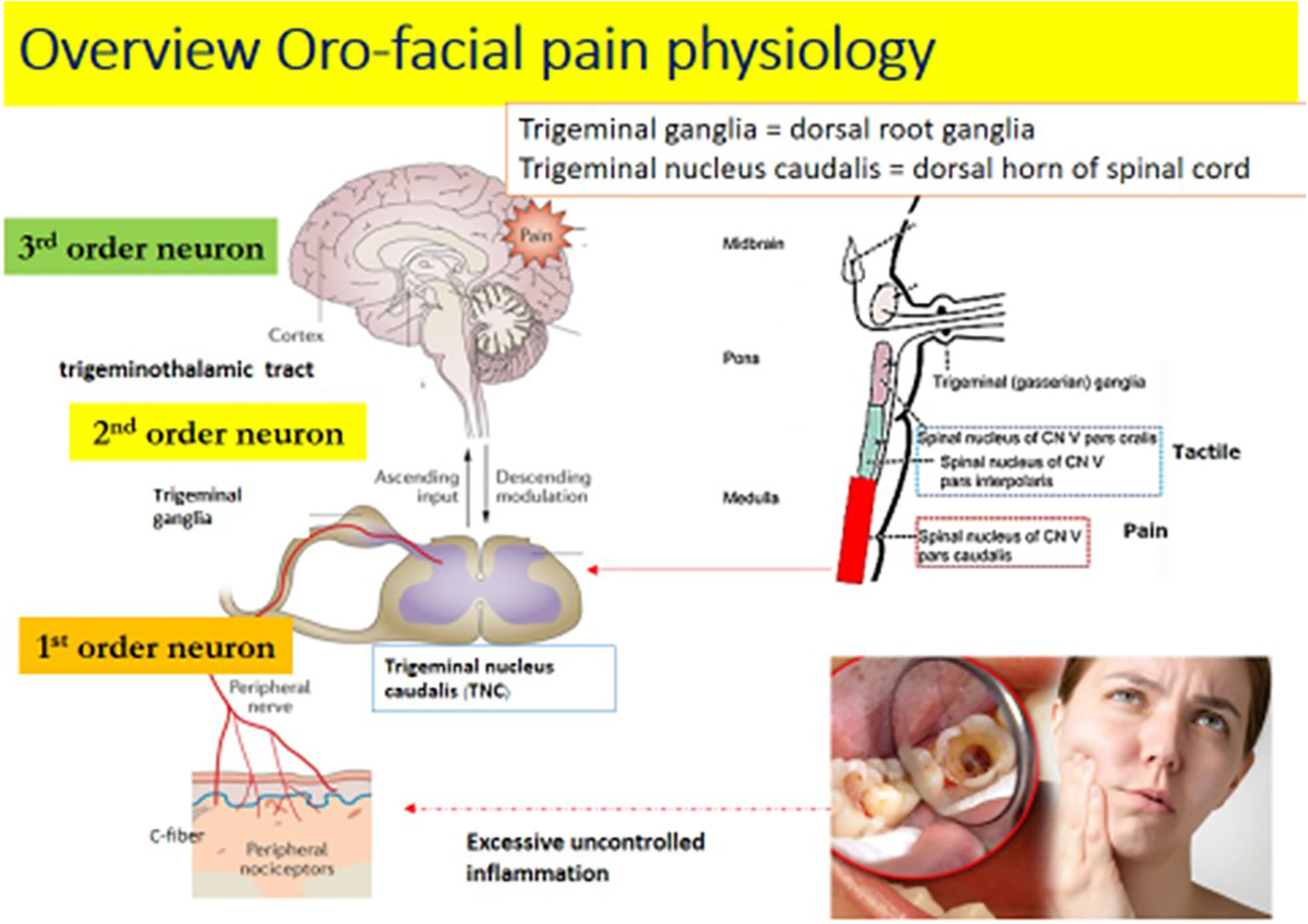
Overview of orofacial pain physiology.

The trigeminal nerve or cranial nerve V (CN V) is a sensory nerve which innervates this region. The trigeminal pain pathway from the orofacial area is represented in Figure 1. At the peripheral nociceptors in the orofacial region, after receiving repetitive noxious stimuli or an excessive uncontrollable inflammation, the first order neurons in the trigeminal nerve will develop increased pain signals that are projected to the trigeminal ganglia (Huff and Daly, 2020). The trigeminal ganglia is similar to the dorsal root ganglia (Armstrong and Herr, 2020).

## Brainstem Nociceptive Neurons

After that, the pain signals are sent to the second order neurons in the trigeminal nucleus caudalis located in the brainstem. The trigeminal nucleus caudalis is similar to the dorsal horn of the spinal cord and contains second order neurons.

For the trigeminal nucleus, there are three groups of nuclei located inside the brainstem (Joseph, 1982; Rivera-Morales, 1986; John, 1990; Wilkinson, 2014). The first spinal nucleus of the CN V is the pars oralis and the second is the par interpolaris. Both of them convey a tactile sensation in the orofacial area. The third spinal nucleus of the CN V is the pars caudalis or trigeminal nucleus caudalis (Christoforou, 2018; Klasser et al., 2018) which carries the pain perception of this involved area. Then the signals will be further projected to the third order neurons in the thalamus via the ventral trigeminothalamic tract (Joseph, 1982; Rivera-Morales, 1986; John, 1990; Roberts, 1991; van der Bilt et al., 2006; Wilkinson, 2014; Badel et al., 2019).

## Higher Brain Function Regulating Orofacial Nociception

In the everse mechanism, there is a descending pathway or pain modulation process from the somatosensory cortex to the trigeminal nucleus caudalis (Sessle, 1987). Naturally, the brainstem perceives ascending sensory input and sends the signal to the thalamus and cortex respectively (Woolf and American Physiological Society, 2004). However, the neurons of the brainstem can also modulate the pain pathway by sending somatosensory signals through the periaqueductal gray, locus coeruleus and rostral ventromedial medulla. Periaqueductal gray is the central midbrain neurons and modulates pain pathways indirectly via other brainstem nucleus, including the locus coeruleus and rostral ventromedial medulla. The locus coeruleus is composed of noradrenergic neurons which are projected to the trigeminal nucleus caudalis. The rostral ventromedial medulla is a large region of medulla which is placed by serotoninergic neurons (Arendt-Nielsen et al., 2018). The descending pathway sends signals to the trigeminal nucleus caudalis. Either serotonin and norepinephrine are released or enkephalin or opioid peptides are produced: this process leads to pain reduction (Fields, 1987; Espinosa-Sanchez et al., 2020). A clinical study reported that patients experiencing temporomandibular joint pain might have a decrease in neurons on both sides of the brainstem, especially at the rostral ventromedial, which is responsible for descending pain pathways or pain modulation. Therefore, the reduction of neurons in the descending pain modulation might increase pain sensation in patients with painful temporomandibular disorders (Wilcox et al., 2015).

Therefore, numerous studies supported that the pathophysiology of orofacial pain is related to the trigeminal nerve and trigeminal pathway leading to alter pain perception in the relevant area of the somatosensory cortex and associated somatosensory cortex.

## Classification of Orofacial Pain and Possible Mechanisms

**Figure 2.**
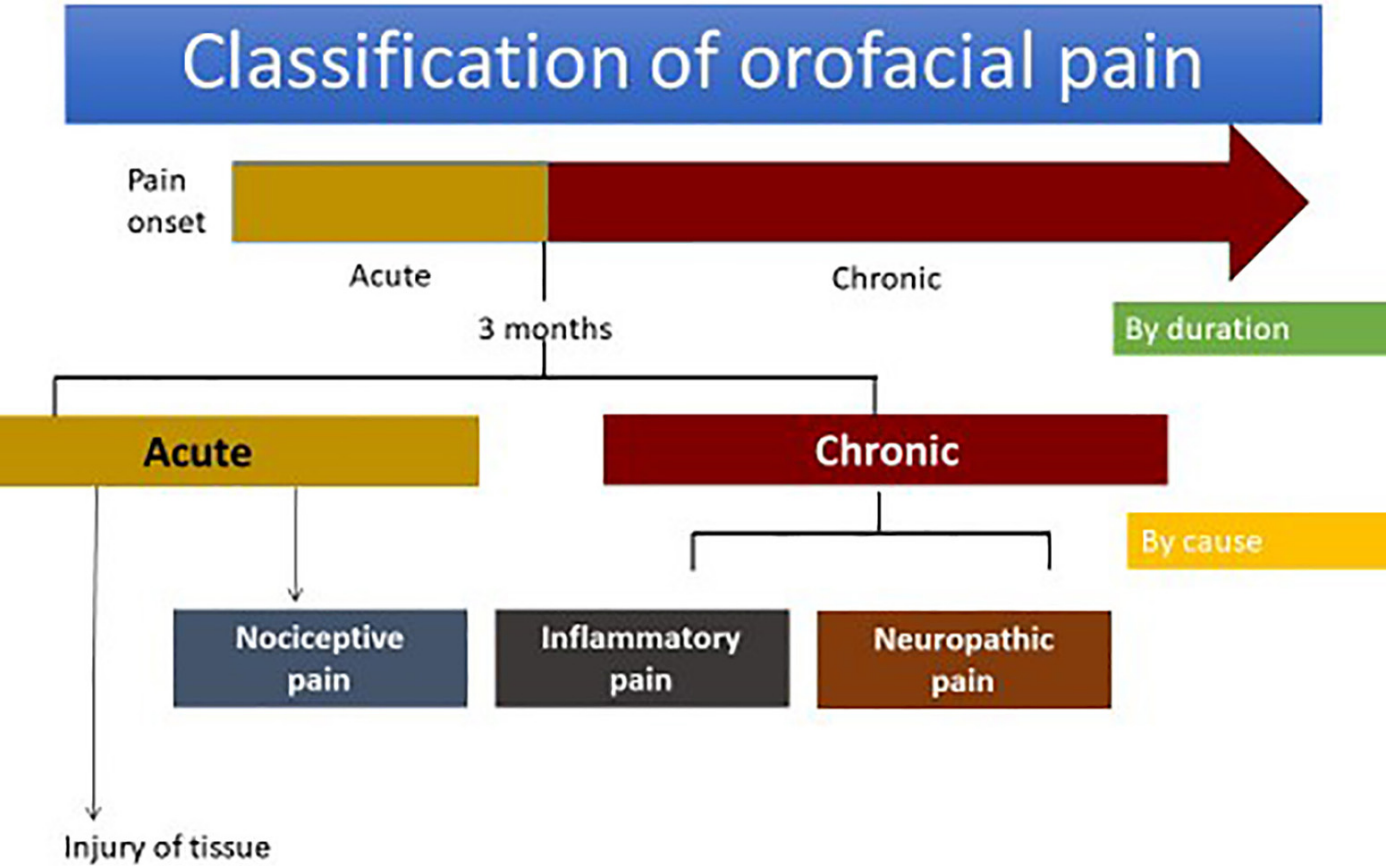
Classification of orofacial pain.

The classification of orofacial pain concerned by durations can be divided into acute pain and chronic pain. By causes, it can be categorized into nociceptive, inflammatory and neuropathic pain (Lee et al., 2005; Fig. 2)

Acute orofacial pain is the sudden onset of pain related to physical sensations and possibly of limited duration as well as being temporary because of tissue injury causes. Chronic orofacial pain is long lasting pain beyond three months, which is the normal healing period of time (Bell, 1989; Buxbaum, 1994; Christopher, 1998; Monheim, 2019). The difference of acute and chronic orofacial pain is shown in Table 1.

**Table 1.** Difference in acute orofacial pain and chronic pain orofacial pain

Characteristics	Acute orofacial pain	Chronic orofacial pain
Duration	Onset	Sustained, persistent >3 months in humans
Cause	Caused by inflammation or injury of tissue	Caused by inflammation, nerve damage and excessive or uncontrolled inflammation
Cause has gone away or healed	No pain when normal healing occurs or is only temporary (pain disappears once stimulus is removed)	Persistent pain and excessive, uncontrolled causes
Signs and symptoms	Sudden, sharp, intense, localized	Aching, diffused
Physiologic response	Acute pain affects increased cardiovascular functions such as increased blood pressure and heart rate via sympathetic response	Chronic pain affects physiological responses with adaptation behaviors or psychological responses such as depression and anxiety
Examples in the orofacial area	(1) Dental pain: pulpitis (2) Mucogingival pain	(1) Neuropathic pain: trigeminal neuralgia, peripheral trigeminal nerve injury, postherpetic neuralgia (2) Chronic inflammatory pain: chronic pulpitis and apical lesions, temporomandibular disorder pain (3) Neurovascular pain: migraines, tension-type headaches

Another classification of orofacial pain is classified by three causative groups; nociceptive, inflammatory and neuropathic pain. Nociceptive pain is pain that occurs when noxious stimuli such as heat, cold, intense mechanical force, and chemical irritants directly stimulate nociceptive sensory neurons. Then the nociceptors send signals to the central nervous system leading to pain response such as withdrawal reflex. Therefore, nociceptive pain mechanisms obviously act as a vital physiological sensation (Ananthan and Benoliel, 2020). Inflammatory pain is a pain caused by damaged tissues. Once tissues are injured, the release of inflammatory mediators subsequently occurs and activates pain perception (Shinoda et al., 2019). Neuropathic pain is a pain caused by defects in the peripheral or central nervous system (Fehér et al., 2019; Jaaskelainen, 2019). Thus, the differences between nociceptive, inflammatory and neuropathic pain are shown in Table 2.

**Table 2.** Difference between nociceptive, inflammatory, and neuropathic pain

Characteristics	Nociceptive orofacial pain	Inflammatory orofacial pain	Neuropathic pain
Causes and mechanism of pain pathway	Noxious stimulation at the peripheral nerve and transmitted by normal components of the sensory trigeminal nerve	Strong noxious stimulus causes lesions in the tissue leading to local inflammation responses and increased inflammatory mediators	Caused by nerve damage or injury and increased peripheral sensitization, structure change by increased sodium activation, calcium activity of nerves leading to ectopic discharges, and glia cell activation
Nerve condition	Normal nerve structure	Normal nerve structure	Abnormal nerve structure
Stimulation	Response to noxious stimulus for protective and withdrawal response	Response to noxious stimulus and increase of activity of peripheral nociceptors	-Response to non-noxious and noxious stimulation -Spontaneous pain without stimulation because ectopic discharges occurred in damaged nerves
Example	Hot soup contacting the oral mucosa immediately caused pain perception (heat/hot), and then they threw away this hot soup	-Pulp necrosis with apical abscess -Temporomandibular joint capsulitis or synovitis is caused by joint inflammation. Joint pain and limitation of jaw movement develops afterward	Peripheral trigeminal nerve injury is caused by nerve damage such as facial trauma accident or trigeminal neuralgia contributing to abnormal nerve structure and expression of severe shooting pain, intermittent patterns, and feels like electric shocks

**Figure 3.**
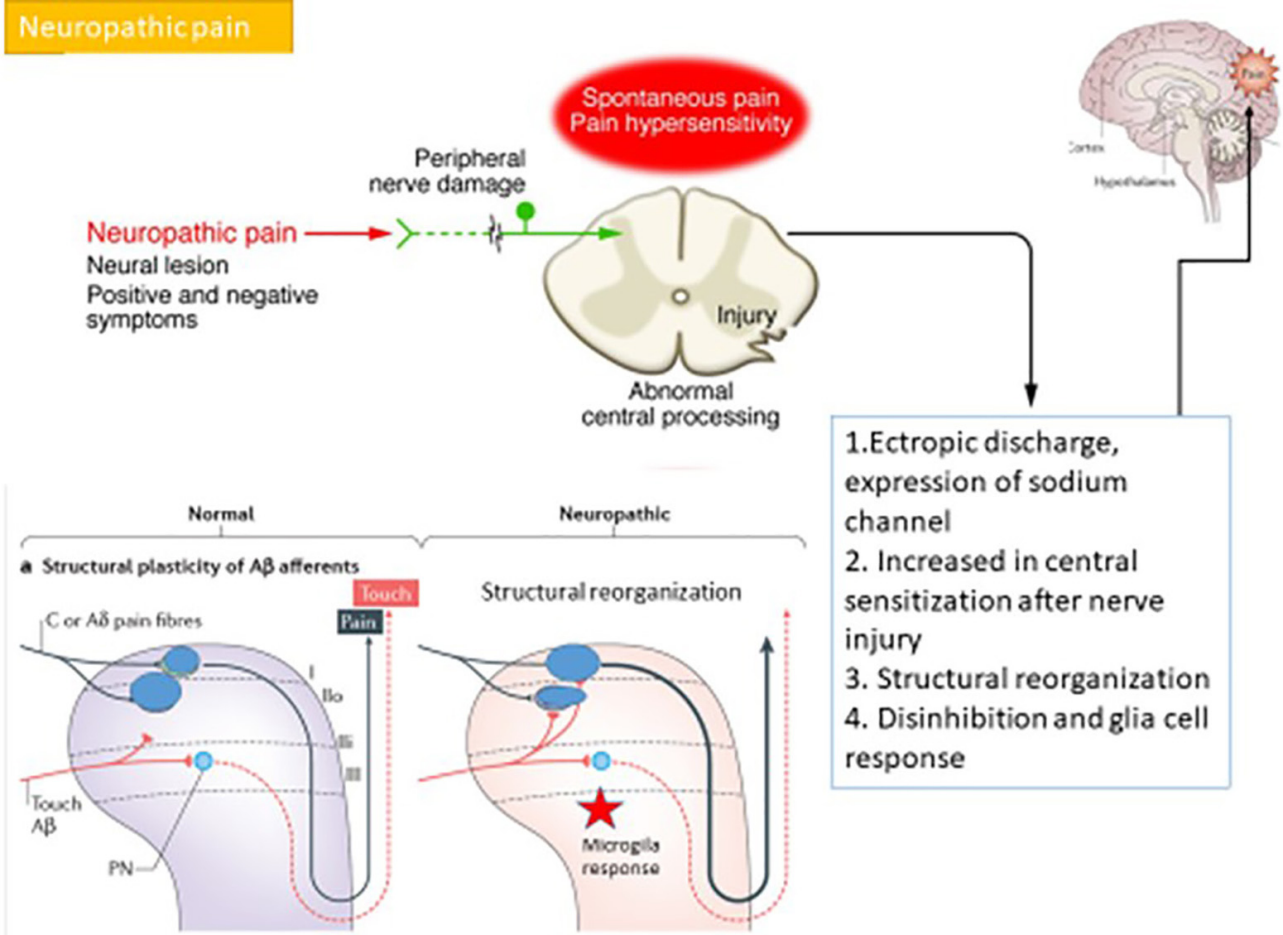
The possible mechanisms of neuropathic pain.

Orofacial neuropathic pain originates from disorders of the somatosensory system. Results of injury to the nerves involved the pain pathway and maybe considered as chronic pain conditions (Jaaskelainen, 2004). The mechanisms of neuropathic pain are distinct from other types of orofacial pain (Fig. 3).

The possible mechanisms of orofacial neuropathic pain are distinguishable from nociceptive and inflammatory pain. After trigeminal nerves are damaged, the neuroma or injured axon around the damaged site proliferate heterotopic sodium channels such as voltage gated channel types 1.3, 1.7, and 1.8 (Allard et al., 2006; Hargus and Patel, 2007). The proliferation of sodium channels may cause lower stimulation thresholds and provoke ectopic discharges (Li et al., 2015). The spread of sodium channels triggers central sensitization at the trigeminal nucleus caudalis and leads to an enhanced barrage of nociceptive signals, which results in spontaneous pain and pain hypersensitivity (Piovesan et al., 2003; Bernal and Roza, 2018).

The alteration of the calcium channel in the primary afferent nociceptors leads to enhanced transmitters which are released into the trigeminal nucleus caudalis (Li et al., 2019). In addition, AMPA receptors activate trigeminal nucleus caudalis neurons that are related to common responses to painful stimuli, but NMDA receptors are physiologically blocked by a magnesium ion. This blockage is likely removed by accumulation or repetitive depolarization from ectopic discharges. Alteration of the calcium channel results in an amplification and prolongation of the noxious input in the trigeminal nucleus caudalis inside the brainstem (Yam et al., 2018; Armstrong and Herr, 2019).

Furthermore, after nerve injury, the peripheral nerve lesion causes axon and myelin sheath degradation which is later followed by infiltration of macrophages and other types of immune cells such as neutrophils and T cells at the injured site (Spencer and Gremillion, 2007; Lebrilla and Mahal, 2009; Todd, 2010). Moreover, nerve damage subsequently generates functional modulation and modification of the central nervous system (Boadas-Vaello et al., 2017).

Functional modulation after nerve injury leads to increased posttranslational processing such as c-fos in the trigeminal nucleus caudalis. Moreover, the coupling between sympathetic postganglionic neurons and afferent neurons under pathophysiological conditions can occur to maintain pain. Modification after nerve injury is altered in connectivity and cell death by some adjacent uninjured nerve fibers which become excited because of non-synaptic or cross talk of electrical transfers called ephaptic transmission. Finally, this modulation and modification function causes an increase in central sensitization in the trigeminal nucleus caudalis and subsequently the recruitment of secondary messenger pathways after central sensitization results in a rise of neuron excitability or nociceptors or pain signals to the thalamus and somatosensory cortex (Yam et al., 2018; Dolphin et al., 2020).

Non-injury state in the area of the trigeminal nucleus caudalis and dorsal horn of the spinal cord, Aβ fibers penetrate to the dorsal horn, travel ventrally and terminate in Lamina II and deeper into the spinal cord. However, after the peripheral nerve is damaged or injured (Nelson, 2019), there is c-fiber terminal atrophy and A fiber terminal sprouting into the superficial dorsal horn, which conducts easier stimulation of pain signals in the dorsal horn of the spinal cord, and is the reason why non-painful stimulators can produce pain responsiveness in allodynia (Song et al., 2013).

In addition, after nerve injury, a loss of inhibition occurs because of dysfunction of GABA production and apoptosis of inhibitory interneurons resulting in reduction of inhibition pain signals (Tashiro et al., 2014). Lack of or a decrease in inhibition pain signals cause an increased excitability of neurons (Yin et al., 2018). Moreover, after prolonged nerve injury, there follows a decrease in pain modulation of descending pain pathways which result in more nociceptive signals in the pain pathway (Sessle, 2011).

Therefore, the possible pain mechanisms related to glia cell response after nerve injury are caused by microglial activation. Microglia can then release pro-inflammatory mediators such as interleukin 6, interleukin 1β, and tumor necrosis factor α, and activate astrocytes (Han et al., 2012; Won et al., 2012; Lemos et al., 2018). Activated astrocytes or reactive astrocytes directly trigger NMDA receptors (Zhou et al., 2019) leading to excess glutamate release to the trigeminal nucleus caudalis and increase pain signaling projected to the brain and development of neuropathic pain (Jancalek, 2011; Mamoru et al., 2011; Eftekhari et al., 2015).

Chronic neuropathic orofacial pain manifests many distinct functions and possible mechanism apart from inflammatory pain (Tandon et al., 2003) Nevertheless, the differential diagnosis should be performed and defined these types of pain because of different pain management (Allegri et al., 2012).

Consequence, if orofacial pain (acute/chronic stages), which is not neuropathic pain, does not produce an ectopic discharge, structural reorganization and glia cell response. This process of inflammatory pain is sustained by chemical inflammation at the primary trigeminal afferents neuron such as substance P, bradykinin, and calcitonin related peptide proteins leading to peripheral sensitization of peripheral nociceptors at the lesion (Tandon et al., 2003; Allegri et al., 2012). However, repetitive stimulation of c-fibers via the inflammation or nerve injury stimulates a gradual increase of neuron excitability or winds them up, causing central sensitization. Evidence showed that blockading NMDA receptors could attenuate chronic inflammatory or neuropathic pain in animal models (Wong et al., 2014; Lin et al., 2019; Neyama et al., 2020). Subsequently, its mechanism can drive central hyper excitability by chemical mediators and other peptides that remove the magnesium-ion block of NMDA receptors at the trigeminal nucleus caudalis, which could possibly increase pain perception (Koichi et al., 2011).

Currently, there is another name of pain classification which called “nociplastic pain.” Nociplastic pain is meant an un-classification of pain or not properly covered by nociceptive pain or neuropathic pain. Therefore, nociplastic pain is caused by an altered nociceptive function but not inflammatory responses. An example of nocociplastic pain is persistent idiopathic dentoalveolar pain, previously known as atypical odontalgia. Persistent idiopathic dentoalveolar pain is a chronic pain condition that shows as a persistent tooth, alveolar bone, gingiva although undetectable pathology during clinical or radiologic examination (Aydede and Shriver, 2018). However, the pathophysiology of nociplastic pain is still unclear, further study about nociplastic pain should be investigated.

## Conclusion

Orofacial pain has become more problematic among the general population. The anatomic complexity of the orofcial region contributes to challenging diagnosis and treatment for many clinicians. A better understanding of underlying physiological mechanisms on orofacial pain may support to improve a clinician’s clarification and perception in the aspect of non-odontogenic or dental pain origin.

## Synthesis

Reviewing Editor: Karen Szumlinski, University of California at Santa Barbara Decisions are customarily a result of the Reviewing Editor and the peer reviewers coming together and discussing their recommendations until a consensus is reached. When revisions are invited, a fact-based synthesis statement explaining their decision and outlining what is needed to prepare a revision will be listed below. The following reviewer(s) agreed to reveal their identity: Yuichi Ogino.

After consultation with two expert reviewers, we have reached the decision of Revise-Rereview. This decision is based primarily on the comments of Reviewer 1, who expressed concerns regarding the organization of the review and the level of detail in each subsection. As no experiments are required, two months should be sufficient to revise the report. Should you require any extra time, please inform eNeuro and we can extend the time. Please find below the specific comments of both reviewers.

Reviewer 1:

n the present review author tried to rereviews the literature to have the better understanding of the Physiology of Orofacial Pain in dentistry.

Major:

1. Author tried to present the review on the Physiology of Orofacial Pain in dentistry. However, author must the describe the strategy for the search ie search terms...... Year of search ie 1990- 20121.

2. Authors must present the thorough review of the topic.

One of the major comment is that, there are no defined sections in the review to present the better understanding of the topic, to understand the central pathway or peripheral pathway or any markers what's latest and specific receptors are involved. For example:

Primary Afferent Neurons

Pathological Changes in Trigeminal Ganglion

Brainstem Nociceptive Neurons

Receptor Mechanisms of Trigeminal Primary Afferent Neurons

Higher Brain Function Regulating Orofacial Nociception

3. Authors are suggested to refer to few of the articles

J. Oral Biosci. 53（4）：318－329, 2011; Indian J Physiol Pharmacol 2003; 47 (3) : 247-269; Journal of Clinical Neurophysiology: November 2019 - Volume 36 - Issue 6 - p 422-429; International Review of Neurobiology Volume 97, 2011, Pages 227-250

Reviewer 2

This review manuscript is an easy-to-understand in pain field in dentistry with comprehensible figures and tables, that will help every dentist in clinical practice.

Minor:

Insert Abbreviation of cranial nerve at the first appearance: (CN).

Major:

As a review literature regarding orofacial pain in dentistry, it would be preferable to refer nociplastic pain (Aydede and Shriver. PAIN 2018). My recommendation is adding a short reference to the nociplastic pain in chronic pain section in your own manner, for example, PIDAP (persistent idiopathic dentoalveolar pain; 6.3 in International Classification of Orofacial Pain, 1st edition) previously known as atypical odontalgia.

## Author Response

Response to reviewers

We thanks to raising point of two reviewers and revised followed these

recommendations

Reviewer 1:

the present review author tried to rereviews the literature to have the better

understanding of the Physiology of Orofacial Pain in dentistry.

Major:

1. Author tried to present the review on the Physiology of Orofacial Pain in

dentistry. However, author must the describe the strategy for the search ie

search terms...... Year of search ie 1990- 2021.

Response: We have added this points in page 1, paragraph 1 of subheading

introduction

2. Authors must present the thorough review of the topic.

One of the major comment is that, there are no defined sections in the review to

present the better understanding of the topic, to understand the central pathway

or peripheral pathway or any markers what's latest and specific receptors are

involved. For example:

Primary Afferent Neurons

Pathological Changes in Trigeminal Ganglion

Brainstem Nociceptive Neurons

Receptor Mechanisms of Trigeminal Primary Afferent Neurons

Higher Brain Function Regulating Orofacial Nociception

Response: We have changes followed the recommendation in blue color in text.

3. Authors are suggested to refer to few of the articles

J. Oral Biosci. 53（4）：318－329, 2011; Indian J Physiol Pharmacol 2003;

47 (3) : 247-269; Journal of Clinical Neurophysiology: November 2019 -

Volume 36 - Issue 6 - p 422-429; International Review of Neurobiology

Volume 97, 2011, Pages 227-250

Response: We have changes followed the recommendation in blue color in text2

Reviewer 2

This review manuscript is an easy-to-understand in pain field in dentistry with

comprehensible figures and tables, that will help every dentist in clinical

practice.

Minor:

Insert Abbreviation of cranial nerve at the first appearance: (CN).

Response: We insert abbreviation of cranial nerve at the first appearance in

page II, paragraph II (red color in text)

Major:

1. As a review literature regarding orofacial pain in dentistry, it would be

preferable to refer nociplastic pain (Aydede and Shriver. PAIN 2018). My

recommendation is adding a short reference to the nociplastic pain in chronic

pain section in your own manner, for example, PIDAP (persistent idiopathic

dentoalveolar pain; 6.3 in International Classification of Orofacial Pain, 1st

edition) previously known as atypical odontalgia.

Response: We thank to raising points. We have added in page 5 paragraph 3

(red color in text)
